# Target receptor expression dictates the selective intra-tumoral targeting of CD8^+^ T cells by eciskafusp alfa in matched PBMCs and TILs from CPI-naïve patients

**DOI:** 10.3389/fimmu.2026.1843841

**Published:** 2026-06-01

**Authors:** Amrita Manchala, Eleni Maria Varypataki, Jehad Charo, Pablo Umaña, Christian Klein, Laura Codarri Deak

**Affiliations:** 1 Roche Pharma Research and Early Development, Roche Innovation Center Zürich, Schlieren, Switzerland; 2 Institute of Experimental Immunology, University of Zurich, Zürich, Switzerland; 3 Department of Biochemistry, Faculty of Chemistry and Pharmacy, Ludwig Maximilians University of Munich, Munich, Germany

**Keywords:** cis-targeting, eciskafusp alfa, IL-2R agonism, immunocytokine, preferential targeting, receptor density, stem-like CD8+ T cells, tumor-infiltrating lymphocytes

## Abstract

**Introduction:**

Immune checkpoint inhibitors (ICIs) targeting the PD-1/PD-L1 axis have shown considerable promise as a therapeutic modality in oncology. Despite their ability to target stem-like CD8^+^ T cells and give rise to exhaustion-fated effector CD8^+^ T cells, a significant subset of patients do not respond or eventually develop resistance, highlighting the need for more efficacious therapies. Eciskafusp alfa (PD1-IL2v) is a novel immunocytokine, engineered for avidity-driven, *cis*-delivery of IL-2R agonism to PD-1^+^ cells.

**Methods:**

This study provides a comprehensive *ex-vivo* characterization of PD1-IL2v’s target landscape using matched peripheral blood mononuclear cells (PBMCs) and tumor-infiltrating lymphocytes (TILs) from patients across seven solid tumor indications.

**Results:**

We confirmed that the TIL compartment is significantly enriched with both stem-like CD8^+^ T cells and immunosuppressive regulatory T cells (Tregs). Notably, PD-1 receptor density was increased up to three-fold on CD8^+^ TILs compared to PBMCs, establishing the basis for preferential intra-tumoral targeting. *Ex-vivo* assays demonstrated that PD1-IL2v preferentially targets CD8^+^ TIL subsets (stem-like and effector) over Tregs. This preferential targeting translated into superior biological activity, with PD1-IL2v inducing higher STAT5 phosphorylation (STAT5-P) in stem-like and effector CD8^+^ T cells compared to Tregs, confirming the intended *cis*-targeting and enhanced IL-2R agonism.

**Discussion:**

These findings provide translational validation for PD1-IL2v’s mechanism, demonstrating selective intra-tumoral immune stimulation while minimizing Treg activation. This characterization identifies PD-1 receptor density and subset prevalence as critical factors for drug activity and represents potentially useful biomarkers for predicting patient responsiveness and guiding patient selection.

## 1 Introduction

Immune checkpoint inhibition, notably PD-1/PD-L1 blockade, has shown promise as a therapeutic modality in oncology, driving durable responses in a considerable subset of patients (1–6). The efficacy of these immune checkpoint inhibitors (ICIs) is measured by their ability to “rescue” CD8^+^ T cells from a state of exhaustion and dysfunction, by blocking the inhibitory PD-1 receptor, promoting T cell proliferation and restoring effector function (6–8). Recent seminal studies have highlighted the role of stem-like CD8^+^ T cells as the key subset mediating response to PD-1 blockade and fueling sustained anti-tumor T cell responses (9–13).

Stem-like CD8^+^ T cells are uniquely characterized by their superior self-renewal and proliferative potential as well as their capacity to give rise to exhaustion-fated effector cells, essential for tumor cell killing (7, 8, 10). Phenotypically, stem-like CD8^+^ T cells co-express PD-1 and the transcription factor T-cell factor 1 (TCF-1). While PD-1 signaling plays a role in preserving this population by attenuating TCR/CD28 co-stimulation and repressing its terminal differentiation (1, 14), TCF-1 is essential for maintaining its “stem-like” properties (8, 15). Interestingly, stem-like T cells are located within specific niches, such as tumor-draining lymph nodes (TdLNs) and tertiary lymphoid structures (TLSs) within the tumor. These “protective” niches are crucial for their persistence, sequestering these cells from excessive inflammatory stimuli (11).

Despite the clinical success of ICIs, a significant proportion of patients do not respond or eventually develop resistance, highlighting the need for more potent and targeted therapies (16–20). Alternative immunotherapeutic strategies, such as cytokine therapy, have also demonstrated enhanced T cell-mediated anti-tumor responses, but face their own distinct set of limitations. Standard IL-2 based therapies, such as high dose IL-2 (HD IL-2) have shown promise but are limited by severe toxicity, owing to on-target, off-tumor binding, mainly due to the pleiotropic expression of IL-2 receptor α (CD25), which together with the β and γ subunits, form a high-affinity receptor (21–24).

To overcome these challenges, PD1-IL2v was developed, consisting of a bivalent PD-1 blocking antibody fused at the Fc-region to an engineered IL2 variant (IL2v), devoid of binding to CD25, thus avoiding its engagement with immunosuppressive regulatory T cells (Tregs), which constitutively express CD25, as well as pulmonary endothelial cells, responsible for vascular leak syndrome associated with IL-2 therapy (21). PD1-IL2v is designed to deliver IL-2 signaling in *cis* to the intermediate affinity IL-2Rβγ (CD122/CD132) receptor, whilst blocking PD-1 signaling (13, 25, 26). Notably, preclinical studies have elegantly demonstrated PD1-IL2v’s ability to target stem-like CD8^+^ T cells, and to induce their differentiation into a phenotypically and functionally unique subset of “better effector” CD8^+^ T cells, resulting in superior anti-tumor efficacy compared to αPD-1 alone (13, 26–28).

The underlying premise of PD1-IL2v’s mechanism of action (MoA) is the concept of avidity-driven selectivity (29), whereby PD1-IL2v preferentially targets PD-1^hi^ subsets which are mainly found in tumors. Preclinical studies using PD1-IL2v have confirmed its targeting and expansion of tumor antigen-specific CD8^+^ TILs, whilst sparing unfavorable Treg expansion (13). Additionally, preclinical studies have also allowed for the characterization of the immune landscape in different tumor models, deepening our understanding of PD1-IL2v’s target tumor microenvironment (TME) profile. The critical translational challenge remains to confirm whether this avidity-driven PD1-IL2v activity holds true in human malignancies.

In this study, we provide a comprehensive *ex-vivo* characterization of the target landscape for PD1-IL2v in matched patient PBMCs and TILs across seven cancer indications. We first assessed the prevalence of key PD-1^+^ subsets in the TME and determined the frequencies and receptor densities of PD-1 and CD122 on these subsets, providing an overview of the expression of PD1-IL2v target receptors between and within PBMC and TIL compartments. Finally, we evaluate the *ex-vivo* targeting and biological activity of PD1-IL2v using STAT5 phosphorylation (STAT5-P) as a readout to confirm not only its preferential targeting on stem-like CD8^+^ T cells, but also the superior signaling response on these targeted populations.

These findings demonstrate the increased biological activity of PD1-IL2v in the tumor compared to the periphery, supporting a mechanism of action that favors local, intra-tumoral immune stimulation, whilst sparing immunosuppressive Tregs. Additionally, an overview of the immune contexture amongst patients and between cancer indications provides a framework for identifying potential indications and patients who may benefit most from this immune-targeted therapeutic approach.

## 2 Materials and methods

### 2.1 PBMCs and DTCs samples acquisition and culturing

Cryopreserved, matched human peripheral blood mononuclear cells (PBMCs) and dissociated tumor cells (DTCs) were purchased from Discovery Life Sciences (Huntsville, AL). The samples were thawed and centrifuged to remove the freezing medium. Cells were resuspended in RPMI 1640 (Gibco™, 21870076) supplemented with 10% Fetal Bovine Serum (FBS) (Gibco™, 26140079) + 1% P/S (Gibco™, 15140122). All samples were then rested at 37 °C for 3–4 hours to allow for recovery and re-expression of thermosensitive surface markers prior to assay initiation.

### 2.2 Receptor quantification

Absolute quantification of PD-1 and CD122 receptor density was performed using the BD Quantibrite™ PE Kit (BD, 340495) according to the manufacturer’s instructions. A standard curve was generated using the Quantibrite beads to convert PE fluorescence intensity (MFI) into PE molecules bound per cell. Using known ratios of PE to antibodies, PE molecules bound per cell were converted to antibodies per cell (ABC).

Following the resting period, single cell suspensions of PBMCs and TILs (ca. 10^5^ cells/well) were first stained with LIVE/DEAD™ Fixable Near IR (780) Viability Kit (ThermoFisher Scientific, L34993) for 20 minutes at 4 °C. Cells were then washed and stained with PE anti-CD122 (1:50, TU27, BioLegend) or PE anti-PD-1 (1:50, EH12.2H7, BioLegend) for 30 minutes at 4 °C. Following another wash, the cells were stained with the following antibodies: Alexa Fluor^®^ 700 anti-CD45 (1:100, 2D1, ThermoFisher Scientific), BV605 anti-CD3 (1:100, OTK3, BioLegend), BUV496 anti-CD4 (1:100, GK1.5, BD), BUV395 anti-CD8 (1:100, RPA-T8, BD), BV711 anti-CD366 (1:100, F38-2E2, BioLegend), FITC anti-CD127 (1:100, HIL-7R-M21, BD). Cells were washed and subsequently fixed overnight at 4 °C in TF Fix/Perm Buffer (4x, 51-9008100, BD) diluted in TF Diluent Buffer (51-9008101, BD). The following day, cells were washed with TF Perm/Wash Buffer (5x, 51-9008102, BD) and incubated with Rabbit mAb anti-PD-1 (1:100, D4W2J, Cell Signaling Technology^®^) for 1 hour at RT. After washing, cells were stained with a secondary BV421-conjugated goat anti-rabbit IgG antibody (1:100, Polyclonal clone, BD) for 30 minutes at 4°C. Finally, cells were stained with Alexa Fluor^®^ 700 anti-TCF1/TCF7 (1:100, C63D9, Cell Signaling Technology^®^) and PE/Dazzle™ 594 anti-FOXP3 (1:50, 206D, BioLegend) for 1 hour at RT.

Note: For the absolute quantification of PD-1 and CD122 receptors, a minimum threshold of 5 events per gated T cell subset was required for inclusion in the analysis. Samples falling below this threshold were excluded to ensure the reliability of gMFI and to prevent bias from stochastic signaling in sparse populations.

### 2.3 Preferential targeting

Following the resting period, PBMCs and TILs (ca. 10^5^ cells/well) were plated in a 96 well U-bottom plate and were exposed to sub-saturating EC50 (0.1 µg/ml) concentrations of PD1-IL2v and FAP-IL2v for 30 minutes at RT. Unbound drug was removed via centrifugation, and cells were then stained with the detection αPGLALA – PE antibody (1:500) and incubated for 30 minutes at 4 °C to detect bound PD1-IL2v or FAP-IL2v. The samples were subsequently stained with LIVE/DEAD™ Fixable Near IR (780) Viability Kit (ThermoFisher Scientific, L34993) for 20 minutes at 4 °C. Surface staining was performed for 30 minutes at 4°C using the following antibodies: Alexa Fluor^®^ 700 anti-CD45 (1:100, 2D1, ThermoFisher Scientific), BV605 anti-CD3 (1:100, OTK3, BioLegend), BUV496 anti-CD4 (1:100, GK1.5, BD), BUV395 anti-CD8 (1:100, RPA-T8, BD), BV711 anti-CD366 (1:100, F38-2E2, BioLegend), FITC anti-CD127 (1:100, HIL-7R-M21, BD). Cells were washed and subsequently fixed overnight at 4 °C in TF Fix/Perm Buffer (4x, 51-9008100, BD) diluted in TF Diluent Buffer (51-9008101, BD). The following day, cells were washed with TF Perm/Wash Buffer (5x, 51-9008102, BD) and incubated with Rabbit a mAb anti-PD-1 (1:100, D4W2J, Cell Signaling Technology^®^) for 1 hour at RT. After washing, cells were stained with a secondary BV421-conjugated goat anti-rabbit IgG antibody (1:100, Polyclonal clone, BD) for 30 minutes at 4°C. Finally, cells were stained with Alexa Fluor^®^ 700 anti-TCF1/TCF7 (1:100, C63D9, Cell Signaling Technology^®^) and PE/Dazzle™ 594 anti-FOXP3 (1:50, 206D, BioLegend) for 1 hour at RT.

### 2.4 STAT5-P assay

Cryopreserved healthy donor (HD) PBMCs (1x10^6^ cells/sample), were resuspended in PBS and plated in a 96 well U-bottom plate (50 μl/well). The cells were first stained with LIVE/DEAD™ Fixable Near IR (780) Viability Kit (ThermoFisher Scientific, L34993) for 20 minutes at 4 °C. Following a 15-minute incubation at 37°C, 100 µl of TF Fix/Perm Buffer (4x, 51-9008100, BD) diluted in TF Diluent Buffer (51-9008101, BD) was added. The samples were incubated for 45 min at RT, before being washed 2 times with TF Perm/Wash Buffer (5x, 51-9008102, BD). Next, the samples were stained with the following surface and intracellular markers: BUV395 anti-CD45 (1:100, HI30, BD), BUV805 anti-CD3 (1:100, SK7, BD), BUV496 anti-CD4 (1:100, SK3, BD), BV711 anti-CD8 (1:100, SK1, BioLegend), FITC anti-PD1 (IC) (1:10, D4W2J, in house-Roche labeling), PE anti-TCF1 (1:100, C63D9, Cell Signaling). After a 1 hour incubation at RT, cells were washed and fixed with BD Phosflow™ Perm Buffer III (558050, BD) for 15 minutes at 4 °C. The samples were washed and stained with PE-CF594 anti-STAT5P (1:100, BD) and APC anti-FoxP3 (1:20 150D, BD) for 1hour at RT.

### 2.5 Data acquisition and analysis

Finally, all samples were washed and re-suspended in 200 µl of FACS Buffer and acquired using a BD FACSymphony A5 instrument using FACSDiva (v9.1; BD Biosciences). Data were subsequently analyzed using FlowJo (v10.8.1; BD Biosciences).

### 2.6 Statistical analyses

For comparisons between two independent groups (e.g., PBMCs vs. TILs within a specific tumor indication), a nonparametric Mann-Whitney U test was performed. For comparisons involving three or more cell subsets within the same indication (e.g., Tregs vs. stem-like CD8^+^ vs. effector CD8^+^), a nonparametric Kruskal-Wallis test was performed. These nonparametric methods were selected to account for the inherent biological variability of patient-derived samples. *Post-hoc* pairwise comparisons were subsequently conducted using Dunn’s multiple comparisons test. Data are presented as mean ± SEM, and a p-value < 0.05 was considered statistically significant. Significance levels are indicated as follows: *p < 0.05, **p < 0.01, ***p < 0.001, and ****p<0.0001.

### 2.7 Illustrations

Figure illustrations were created using BioRender.com.

## 3 Results

### 3.1 PD-1^+^ T cell subsets are enriched in the tumor

**Figure 1:**
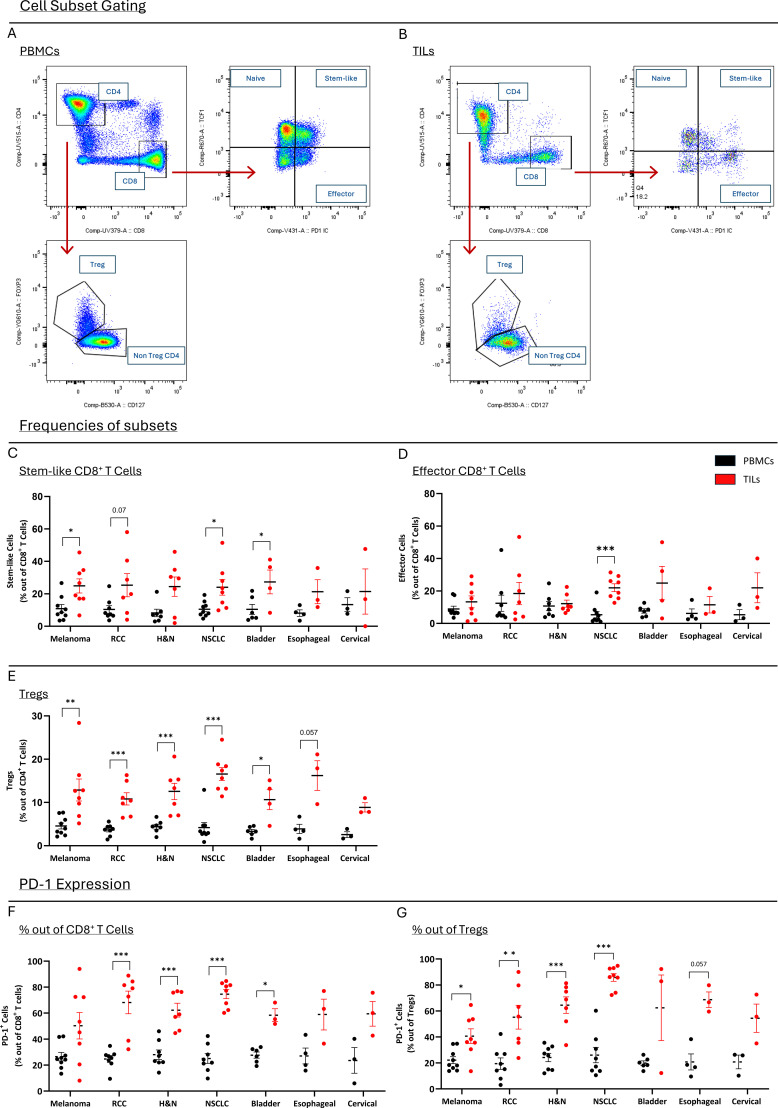
Characterization and enrichment of T cell subsets in patient material **(A, B)** representative flow cytometry gating strategy used to define T cell subsets in matched patient **(A)** PBMCs and **(B)** TILs. **(C, D)** frequency of **(C)** stem-like CD8^+^ T cells and **(D)** effector CD8^+^ T cells as a percentage of total CD8^+^ T cells. **(E)** frequency of immunosuppressive Tregs as a percentage of total CD4^+^ T cells. **(F, G)** frequency of total PD-1^+^ cells within **(F)** CD8^+^ T cell compartment and **(G)** Treg compartment. Statistical significance between PBMCs (black) and TILs (red) for each indication was determined by nonparametric Mann-Whitney U tests. Horizontal lines represent the mean ± SEM; each dot represents an individual patient sample. *p < 0.05, **p < 0.01, ***p < 0.001, and ****p < 0.0001.

We used flow cytometry to characterize T cell subsets in matched PBMCs and TILs acquired from CPI-naïve cancer patients across seven indications (**Figures 1A, B**). Key subsets were defined based on well-established markers, following which the CD8^+^ T cell fraction was subdivided into Naïve (TCF-1^+^, PD-1-), Stem-like (TCF-1^+^, PD-1^+^) and Effector (TCF-1^-^, PD-1^+^) T cells, and CD4^+^ T cells into Tregs (CD4^+^, FoxP3^+^, CD127^-^) and non-Treg (CD4^+^ FoxP3^-^) T cells (**Supplementary Figure 1A**). Healthy donor PBMCs were also processed and analyzed as a reference control (**Supplementary Figure 1B**).

We first quantified the composition of CD8^+^ subsets relative to their parent populations. Stem-like CD8^+^ T cells, relative to total CD8^+^ T cells, were significantly more prevalent in the TIL compartment, notably in melanoma, non-small cell lung cancer (NSCLC) and bladder cancer patients, where we observed significantly higher frequencies (**Figure 1C**). Effector CD8^+^ T cells, relative to total CD8^+^ T cells, were present at more comparable levels between compartments, with significantly higher frequencies of effector CD8^+^ TILs found only in NSCLC patients (**Figure 1D**). As expected, we observed a significant enrichment in the frequency of immunosuppressive Tregs, relative to CD4^+^ T cells, in the TIL compartment compared to matching PBMCs across all indications except cervical cancer (**Figure 1E**). When comparing these subset frequencies from the peripheral blood of patients to healthy donor PBMCs, we found them to be comparable, suggesting that disease status did not significantly alter the prevalence of these populations in the periphery (**Supplementary Figure 1C**).

In order to compare across T cell subsets, we quantified the frequencies of all key subsets relative to the total CD3^+^ T cell population. These findings confirmed that the TIL compartment contains higher frequencies of Tregs (with significant increases in melanoma, H&N and NSCLC patients) compared to the periphery (**Supplementary Figure 1D**). We also observed significantly higher frequencies of stem-like CD8^+^ T cells in bladder and NSCLC patients, with significantly higher frequencies of effector CD8^+^ T cells in the latter indication (**Supplementary Figures 1E, F**).

Next, to be able to understand and predict binding behavior of PD1-IL2v, we measured the expression of PD-1 on these subsets. The frequency of PD-1^+^ cells within the CD8^+^ T cell compartment was enriched in TILs compared to matched PBMCs across multiple indications, with significant increases observed in RCC (24.71% vs 68.24%), H&N (28.00% vs 62.31%), NSCLC (25.03% vs 74.66%) and bladder cancer (27.65% vs 58.40%) (**Figure 1F**). Similarly, PD-1^+^ Tregs were also enriched in the TIL compartment, with significant increases in melanoma (22.10% vs 40.64%), RCC (19.45% vs 55.21%), H&N (24.18% vs 64.49%), NSCLC (26.03% vs 85.75%) and esophageal cancer (20.77% vs 68.67%) (**Figure 1G**). When comparing intra-tumoral PD-1^+^ CD8^+^ T cells versus Tregs relative to total T cells, we observed significantly higher frequencies of PD-1^+^ CD8^+^ T cells compared to PD-1^+^ Tregs in melanoma (5.38% vs 2.40%), RCC (17.68% vs 1.78%), H&N (7.47% vs 3.66%) and NSCLC (14.04% vs 5.37%) patients (**Supplementary Figure 1G**). The direct comparison between healthy donors and cryopreserved patient material revealed an elevated frequency of PD-1^+^ cells within total CD8^+^ and Treg subsets in cancer patients compared to healthy individuals (**Figures 1F, G**) (**Supplementary Figure 1H**).

These data confirm that the TIL compartment represents a therapeutic niche, characterized by the co-enrichment and expansion of both stem-like CD8^+^ TILs as well as the unfavorable immunosuppressive Treg population. This niche is further characterized by a tendency for higher frequencies of PD-1^+^ T cells within the CD8^+^ T cell subset compared to Tregs.

### 3.2 PD-1 receptor enrichment on TILs favors avidity-driven targeting by PD1-IL2v

**Figure 2:**
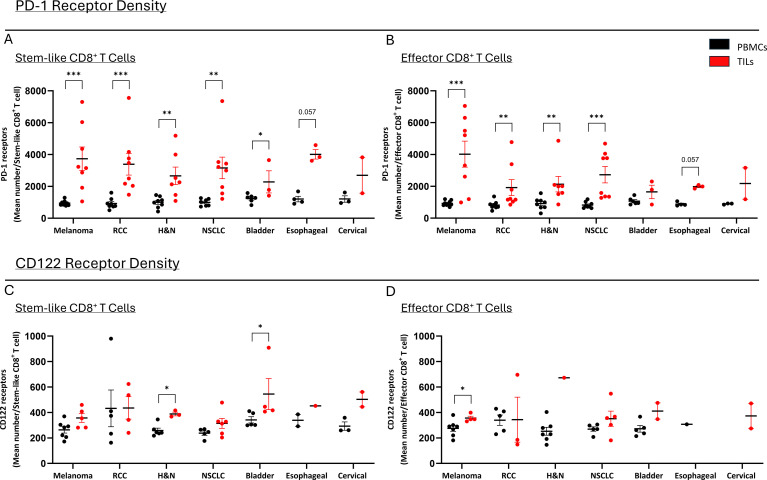
PD-1 receptor density is upregulated on TILs, favoring selective targeting **(A, B)** absolute quantification of PD-1 receptor number on **(A)** stem-like and **(B)** effector CD8^+^ T cells. **(C, D)** absolute quantification of CD122 receptor number on **(C)** stem-like and **(D)** effector CD8^+^ T cells. PBMCs are depicted in black and TILs in red in all graphs. Statistical significance between PBMCs (black) and TILs (red) for each indication was determined by nonparametric Mann-Whitney U tests. Horizontal lines represent the mean ± SEM; each dot represents an individual patient sample. Receptor quantification was only performed for subsets containing at least 5 recorded events to ensure signal reliability. *p < 0.05, **p < 0.01, ***p < 0.001, and ****p < 0.0001.

To understand how PD1-IL2v can achieve tumor selectivity, we quantified the absolute receptor density of its two primary targets, PD-1 and CD122, on stem-like and effector CD8^+^ TILs as well as Tregs. We found that in addition to being enriched in the tumor compartment (**Figure 1C**) (**Supplementary Figure 1E**), stem-like CD8^+^ TILs also expressed higher amounts of PD-1 receptors compared to PBMCs, exhibiting an average three-fold increase between compartments in all indications except esophageal and cervical cancer (**Figure 2A**). In line with these findings, CD8^+^ effector TILs (**Figure 2B**) and Treg TILs (**Supplementary Figure 2A**) also significantly upregulated PD-1 receptor expression on all indications except bladder and cervical cancer. When comparing PD-1 receptor density on total CD8^+^ T cells versus Treg TILs, we observed no significant differences between subsets across all indications (**Supplementary Figure 2B**). However, when focusing on stem-like CD8^+^ T cells, the primary target population of PD1-IL2v, we observed a consistent trend towards higher PD-1 receptor amounts compared to Tregs across most indications, with significantly higher amounts in melanoma patients (**Supplementary Figure 2C**).

In contrast, when assessing CD122 density on these same subsets, unlike PD-1, we observed largely comparable receptor expression between compartments (**Figure 2D**) (**Supplementary Figure 2D**). Significant increases in CD122 receptor density were observed on stem-like CD8^+^ T cells in H&N and bladder cancer patients (**Figure 2C**), on effector CD8^+^ T cells in melanoma patients (**Figure 2D**) and on Tregs in NSCLC patients (**Supplementary Figure 2D**). Interestingly, CD122 expression was on average ten-fold lower than PD-1 across both subsets and indications, while a higher frequency of CD122 was observed in PD1^+^ Tregs compared to the expression in PD1^+^ CD8^+^ T cells (**Supplementary Figures 2E, F**).

Collectively, these findings demonstrate the heightened PD-1 receptor density observed across indications on CD8^+^ TILs as opposed to peripheral blood, providing a solid rationale for the tumor-selective targeting properties of PD1-IL2v observed in pre-clinical efficacy studies in mice.

### 3.3 PD-1 and CD122 receptor co-expression validates the target population for PD1-IL2v

**Figure 3:**
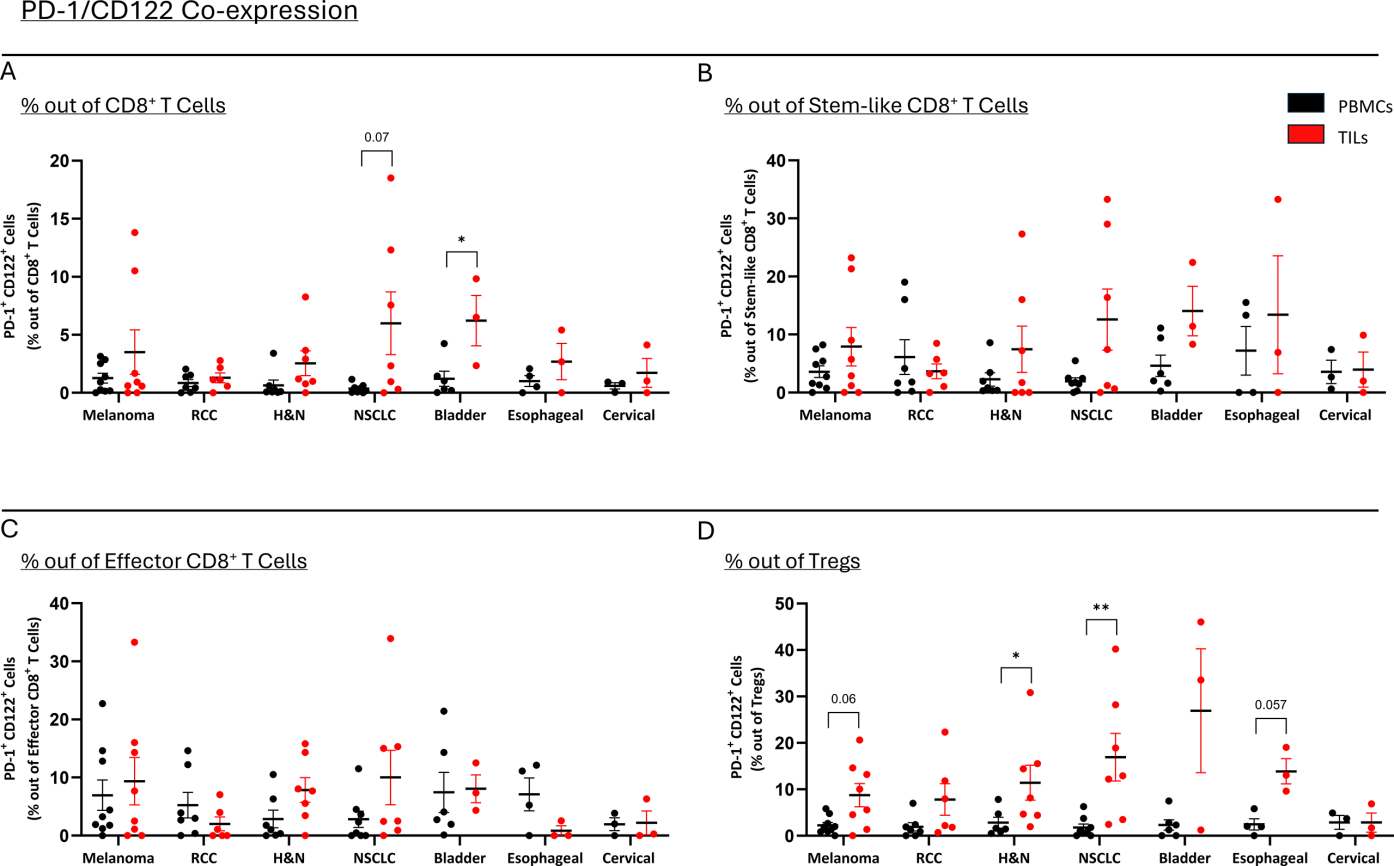
PD-1/CD122 co-expression validates the functionally competent PD1-IL2v-target pool in TILs **(A-D)** frequency of PD-1^+^ CD122^+^ cells out of **(A)** total CD8^+^ T cells, **(B)** stem-like CD8^+^ T cells, **(C)** effector CD8^+^ T cells and **(D)** Tregs. PBMCs are depicted in black and TILs in red in all graphs. Statistical significance between PBMCs (black) and TILs (red) for each indication was determined by nonparametric Mann-Whitney U tests. Horizontal lines represent the mean ± SEM; each dot represents an individual patient sample. *p < 0.05, **p < 0.01, ***p < 0.001, and ****p < 0.0001.

Given that PD1-IL2v is designed to deliver IL-2R agonism in *cis* while blocking PD-1, we assessed the frequency of PD-1^+^ CD122^+^ co-expressing subsets across indications (**Figure 3**).

Our analysis confirmed the presence of PD-1^+^ CD122^+^ co-expressing CD8^+^ T cells in both compartments, with significantly higher frequencies (relative to CD8^+^ T cells) in CD8^+^ TILs in NSCLC and bladder cancer patients (**Figure 3A**). Importantly, we observed a consistent trend towards higher frequencies of PD-1^+^ CD122^+^ stem-like CD8^+^ TILs (relative to stem-like CD8^+^ T cells) compared to PBMCs, especially evident in H&N, NSCLC and bladder cancer patients (**Figure 3B**). This compartment-based difference was less pronounced in effector CD8^+^ T cells (**Figure 3C**). Within the Treg subset, we observed significantly higher frequencies of CD122^+^ PD-1^+^ Treg TILs compared to PBMCs in melanoma, H&N and NSCLC patients (**Figure 3D**).

To assess the overall magnitude of this competent target pool, we also quantified the frequency of PD-1^+^ CD122^+^ subsets relative to the total CD3^+^ T cell population (**Supplementary Figures 3A–D**). This analysis confirmed the presence of co-expressing Tregs and CD8^+^ subsets, with significantly higher frequencies of co-expressing Tregs in the TIL compartment of melanoma, H&N, NSCLC and bladder cancer patients (**Supplementary Figure 3A**). We observed significantly higher co-expressing stem-like CD8^+^ T cells in bladder cancer patients and higher frequencies in many other indications, both within effector and stem-like CD8^+^ T cell subsets, although not significant (**Supplementary Figures 3B, D**).

These findings highlight the critical prevalence of PD-1^+^ CD122^+^ stem-like CD8^+^ TILs, validating that this progenitor pool is IL-2R competent and possesses the necessary targets for optimal PD1-IL2v engagement. However, the presence of co-expressing Tregs in the TME underscores the need for preferential targeting of PD1-IL2v to CD8^+^ T cells to maximize therapeutic benefit.

### 3.4 PD1-IL2v preferentially targets stem-like and effector cells CD8^+^ TILs compared to PBMCs

**Figure 4:**
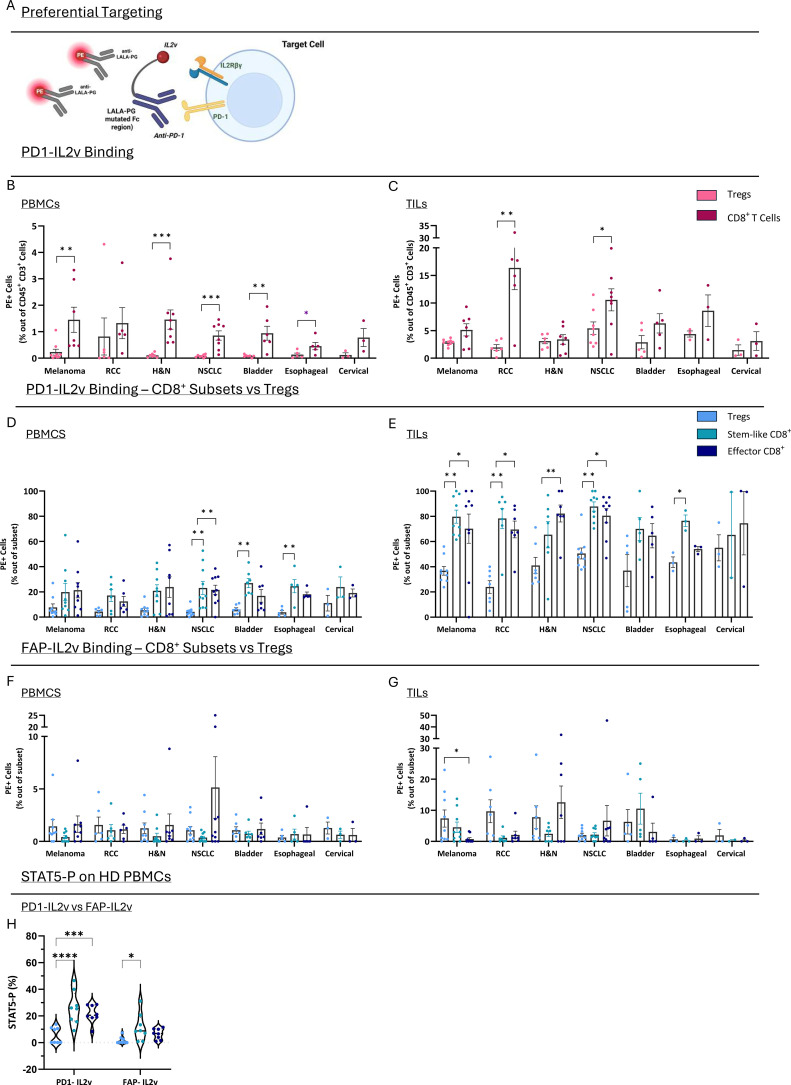
PD1-IL2v achieves intra-tumoral selectivity for CD8^+^ T cell subsets **(A)** schematic illustration of the PD1-IL2v mechanism of action, showing binding to PD-1 and IL-2R and detection via αPGLALA – PE detection antibody. **(B, C)** frequency of PD1-IL2v-bound Tregs (light pink) and total CD8^+^ T cells (purple) out of total CD3^+^ T cells in **(B)** PBMCs and **(C)** TILs. **(D, E)** frequency of PD1-IL2v-bound Tregs (light blue), stem-like (turquoise) and effector (dark blue) CD8^+^ T cells out of their respective subsets in **(D)** PBMCs and **(E)** TILs. **(F, G)** frequency of FAP-IL2v-bound Tregs (light blue), stem-like (turquoise) and effector (dark blue) CD8^+^ T cells out of their respective subsets in **(F)** PBMCs and **(G)** TILs. **(H)** frequency of STAT5-P^+^ cells out of Tregs (light blue), stem-like (turquoise) and effector (dark blue) CD8^+^ T cells upon PD1-IL2v (left) and FAP-IL2v (right) exposure tested on HD PBMCs. Comparisons between total CD8^+^ T cells and Tregs within each compartment were determined by nonparametric Mann-Whitney U tests. Differences across T cell subsets within each indication were determined by a Kruskal-Wallis test followed by Dunn’s multiple comparisons *post-hoc* test. For the STAT5-P analysis in healthy donor PBMCs (h), the same nonparametric approach was applied to compare the three subsets. Horizontal lines represent the mean ± SEM; each dot represents an individual patient sample. *p < 0.05, **p < 0.01, ***p < 0.001, and ****p < 0.0001.

Having established that TILs possess significantly higher PD-1 receptor densities (**Figure 2**) and co-express PD-1 and CD122 (**Figure 3**), we next sought to directly evaluate which subsets PD1-IL2v preferentially targets (**Figure 4A**). Consistent with the PD-1^hi^ TIL signature, PBMCs showed low overall PD1-IL2v binding levels to total CD8^+^ cells and Tregs (<2%) across all subsets and indications (**Figure 4B**), while TILs exhibited significantly higher overall binding (**Figure 4C**). Interestingly, our untargeted FAP-IL2v control demonstrated minimal (<2%) and indiscriminate binding in both PBMCs and TILs (**Supplementary Figures 4A, B**), confirming that PD1-IL2v binding is strongly dependent on the PD-1 targeting mechanism.

We then assessed intra-tumoral selectivity by comparing PD1-IL2v binding to Tregs versus CD8^+^ T cell subsets of interest within the TIL compartment. Firstly, in the periphery, despite lower overall binding compared to the tumor compartment, we still observed preferential targeting of CD8^+^ subsets over Tregs, with significantly higher binding in NSCLC, bladder and esophageal cancer patients (**Figure 4D**). In the TIL compartment, these differences were significantly more striking, with CD8^+^ T cell subsets (both stem-like and effector) showing on average a two-fold increase in binding compared to Tregs (**Figure 4E**), and significantly higher levels of drug-bound subsets compared to their matched peripheral counterparts (**Figure 4D**). When comparing stem-like CD8^+^ TILs to Tregs, we observed significantly higher PD1-IL2v binding in melanoma, RCC, NSCLC and esophageal cancer patients, with consistently higher trends in all other indications as well. Effector CD8^+^ T cells showed a similar pattern of preferential binding of PD1-IL2v as stem-like CD8^+^ TILs over Tregs, with significantly higher binding levels in melanoma, RCC, H&N, and NSCLC patients (**Figure 4E**). This preferential targeting of CD8^+^ subsets over Tregs was not observed in the control group treated with FAP-IL2v, neither between cell subsets nor between cell compartments (**Figures 4F, G**).

These findings validate the tumor tropism of PD1-IL2v and its therapeutic design rationale, highlighting its ability to not only preferentially target the TIL compartment, but also to exhibit intra-tumoral selectivity for CD8^+^ T cell subsets over immunosuppressive Tregs, improving its therapeutic effect.

To confirm that the preferential targeting translates into biological activity, we assessed STAT5-P in HD PBMCs (**Figure 4H**). Treatment with PD1-IL2v induced a specific and robust STAT5-P signal compared to the non-targeted FAP-IL2v control in CD8^+^ T cell subsets. Stem-like CD8^+^ T cells exhibited significantly higher STAT5-P upon PD1-IL2v binding compared to FAP-IL2v (**Supplementary Figure 4C**), confirming that PD-1 targeting improves IL-2R agonism potency. Furthermore, STAT5-P was significantly higher on both stem-like and effector CD8^+^ subsets compared to Tregs upon PD1-IL2v (**Figure 4H**). This data validates that stem-like CD8^+^ T cells are intrinsically PD-1 and IL-2 signaling competent, and that they are preferential targeted by PD1-IL2v as intended for its therapeutic function.

## 4 Discussion

The therapeutic efficacy of PD-1 blockade is shown to rely on its ability to act upon stem-like CD8^+^ T cells, which serve as the progenitor pool needed to sustain a durable T cell-mediated anti-tumor immune response (1, 3, 6, 11). In this study, we analyzed patient-derived PBMCs and TILs from multiple solid tumor indications to provide the first comprehensive *ex-vivo* validation of the targeting mechanism of PD1-IL2v in human cancers.

Our characterization confirmed the criticality of the TIL compartment as a therapeutic niche, defined by the presence of both stem-like CD8^+^ T cells and immunosuppressive Tregs. Importantly, we demonstrated that PD-1 density was significantly (up to three-fold) increased on both CD8^+^ TILs and Tregs compared to their matched PBMC compartment. When comparing PD-1 receptor density on stem-like CD8^+^ TILs to Tregs, we observed a consistent trend toward higher PD-1 receptor amounts on stem-like CD8^+^ TILs across most indications, with a significant density advantage in melanoma patients. Conversely, CD122 showed largely comparable expression between compartments and was expressed at significantly lower levels than PD-1, establishing a crucial basis for PD1-IL2v’s high selectivity towards PD-1^+^ T cells. PD1-IL2v has been designed to be avidity-driven, achieving stable binding to the PD-1^hi^ cells, which we show are predominantly found within the tumor. The minimal and indiscriminate binding observed with the FAP-IL2v control further confirmed that the selective targeting of PD1-IL2v is PD-1-driven.

The ability of PD1-IL2v to minimize Treg stimulation is critical, given that unintended Treg activation is a major limitation of conventional IL-2-based therapies (21, 22, 30, 31). Our findings provide an important translational bridge from pre-clinical models to the clinic, by demonstrating that PD1-IL2v achieves superior intra-tumoral targeting of CD8^+^ T cell subsets over Tregs in patient-derived tumor samples. This preferential targeting is crucial for optimal PD1-IL2v activity and is hypothesized to target and drive the differentiation of stem-like CD8^+^ T cells into functionally superior better effector cells, validating our previous pre-clinical findings (13, 25).

The observed finding that high PD-1 density on stem-like and effector CD8^+^ TILs leads to the preferential binding of PD1-IL2v was functionally demonstrated by the STAT5-P readout, providing direct evidence of ongoing IL-2R signaling. PD1-IL2v induced significantly higher STAT5-P in stem-like and effector CD8^+^ T cell subsets compared to Tregs. This selective functional activity confirms the intended *cis*-targeting mechanism and demonstrates that PD1-IL2v’s superior IL-2R agonism is PD-1 dependent. Furthermore, our results validate the CD8^+^ progenitor pool as the primary target of PD1-IL2v therapy and position STAT5-P as an important surrogate marker for monitoring and predicting downstream enhanced effector function.

Our comprehensive analysis across seven distinct solid tumor indications highlights the profound variability in immune contexture across patient samples and different cancers. The varying degrees of stem-like CD8^+^ T cell enrichment and PD-1 density across tumor types suggest that responsiveness to PD1-IL2v can be predicted based on this baseline target landscape. Typically, tumor indications with a high prevalence of stem-like CD8^+^ T cells and high PD-1 density, such as melanoma and NSCLC, are predicted to be highly responsive to PD-1 blocking therapies. Importantly, the PD-1 density on Tregs is also found to be high in these same indications, as observed in NSCLC. Conversely, indications with lower target density and fewer stem-like CD8^+^ T cells may necessitate combinatorial strategies to enhance T cell infiltration or activation (12, 32–36). However, it is important to acknowledge the complexity of the TME, whereby a favorable tumor immune landscape does not guarantee total efficacy, since therapy outcome is often dictated by the interplay of cell subsets and various pro-tumorigenic mechanisms in the TME (33–42).

Despite providing strong mechanistic validation, our study has limitations. While our setup allowed us to characterize the avidity-driven targeting of PD1-IL2v in human samples, *ex-vivo* settings are static and therefore do not fully recapitulate the dynamicity of the TME *in-vivo*. In addition, although our *ex-vivo* analyses of receptor density and preferential targeting were performed in matched PBMC and TIL samples, the functional STAT5-P readout for the assessment of IL-2R signaling was evaluated solely in healthy donor PBMCs, due to the limited availability of patient material and the high number of cells required for the specific assay. Therefore, our functional data should be regarded as supportive mechanistic evidence and not as definitive confirmation of PD1-IL2v functional selectivity in the TME; for such direct validation, evaluation in cancer patient TILs is needed. Furthermore, future work must directly link this preferential targeting and signaling to tangible downstream outcomes, such as proliferation, differentiation and enhanced cytotoxicity/effector functions of these targeted subsets.

Ultimately, correlating pre-treatment PD-1 receptor density and stem-like CD8^+^ T cell prevalence with clinical outcomes following PD1-IL2v therapy will be essential to fully substantiate the clinical utility of our findings. The metrics defined in our study could potentially be translated into clinical practice, provided that flow cytometry-based quantification is harmonized to routine histology/immunohistochemistry (IHC) diagnostics. Flow cytometry-based quantification must be standardized using calibration beads and appropriate controls to convert fluorescence intensity into absolute receptor copies (per cell). Furthermore, using identical markers and antibody clones to define populations across platforms would allow the flow cytometry readout to be bridged with IHC tissue-scoring assays. These methodologies provide complementary information: histology/IHC preserves the spatial architecture of the TME, depicting the localization of PD-1 and stem-like CD8^+^ T cell markers, whereas flow cytometry provides absolute and specific quantitative metrics regarding abundance and cell type-specific expression. Therefore, integrating both these approaches could better inform potential drug target accessibility and improve patient stratification strategies; however, thorough analytical validation of both assays remains a prerequisite for clinical implementation.

In conclusion, this study provides strong *ex-vivo* validation of the avidity-driven targeting mechanism of PD1-IL2v. We demonstrate that PD1-IL2v successfully leverages the PD-1^hi^ signature of the human TIL compartment to selectively deliver increased IL-2R agonism to the stem-like CD8^+^ T cell subset while sparing intra-tumoral Treg-mediated immune suppression. Our detailed characterization of both the intrinsic composition and absolute abundance of T cell subsets and receptor density across indications directly establishes the basis for using stem-like CD8^+^ T cell prevalence and PD-1 receptor density as informative biomarkers to predict responsiveness and guide patient selection.

## Data Availability

The original contributions presented in the study are included in the article/**Supplementary Material**. Further inquiries can be directed to the corresponding author.
