# Supplementary material for: Target receptor expression dictates the selective intra-tumoral targeting of CD8+ T cells by eciskafusp alfa in matched PBMCs and TILs from CPI-naïve patients
Source: Front Immunol. 2026 Jun 1;17:1843841. doi: 10.3389/fimmu.2026.1843841 (PMC13265463; doi:10.3389/fimmu.2026.1843841)
Supplement: Supplementary file 1 [file SupplementaryFile1.pdf]

Supplementary Figures

Figure S1

A

Cell Subset	Key Markers
CD8+ Naïve (Q1)	*PD-1⁻, TCF-1⁺*
CD8+ Stem-like (Q2)	*PD-1⁺, TCF-1⁺*
CD8+ Effector (Q3)	*PD-1⁺, TCF-1⁻*
Treg	*CD4+ , FoxP3⁺, CD127⁻*

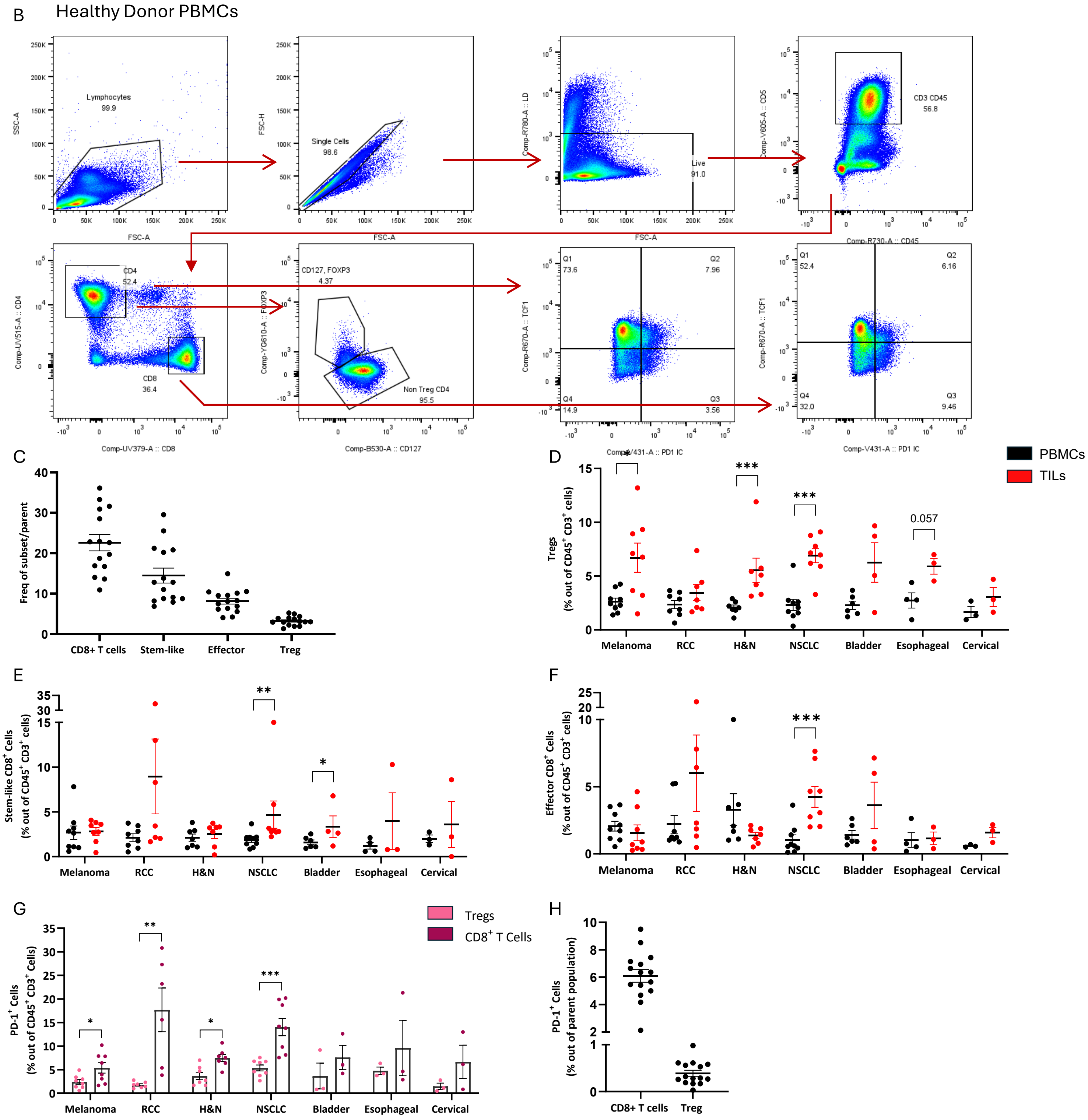

Fig. S1. (A) Table depicting markers used to define key subsets of interest. (B) Flow cytometry plots depicting gating strategy for healthy donor PBMCs. (C) Frequencies of key subsets out of parent population in healthy donor PBMCs. (D-F) Frequency of (D) Tregs, (E) stem-like and (F) effector CD8+ T cells out of total CD3+ T cells in matched patient PBMCs (black) and TILs (red) in all graphs. (G) Frequency of PD-1⁺ CD8⁺ T cells versus Tregs out of total T cells in the TIL compartment. (H) Frequency of PD-1⁺ cells out of CD8⁺ T cells and Tregs in healthy donor PBMCs. Statistical significance between PBMCs (black) and TILs (red) for each indication as well as total CD8⁺ T cells (purple) and Tregs (pink) within each compartment was determined by nonparametric Mann-Whitney U tests. Horizontal lines represent the mean ± SEM; each dot represents an individual patient sample.

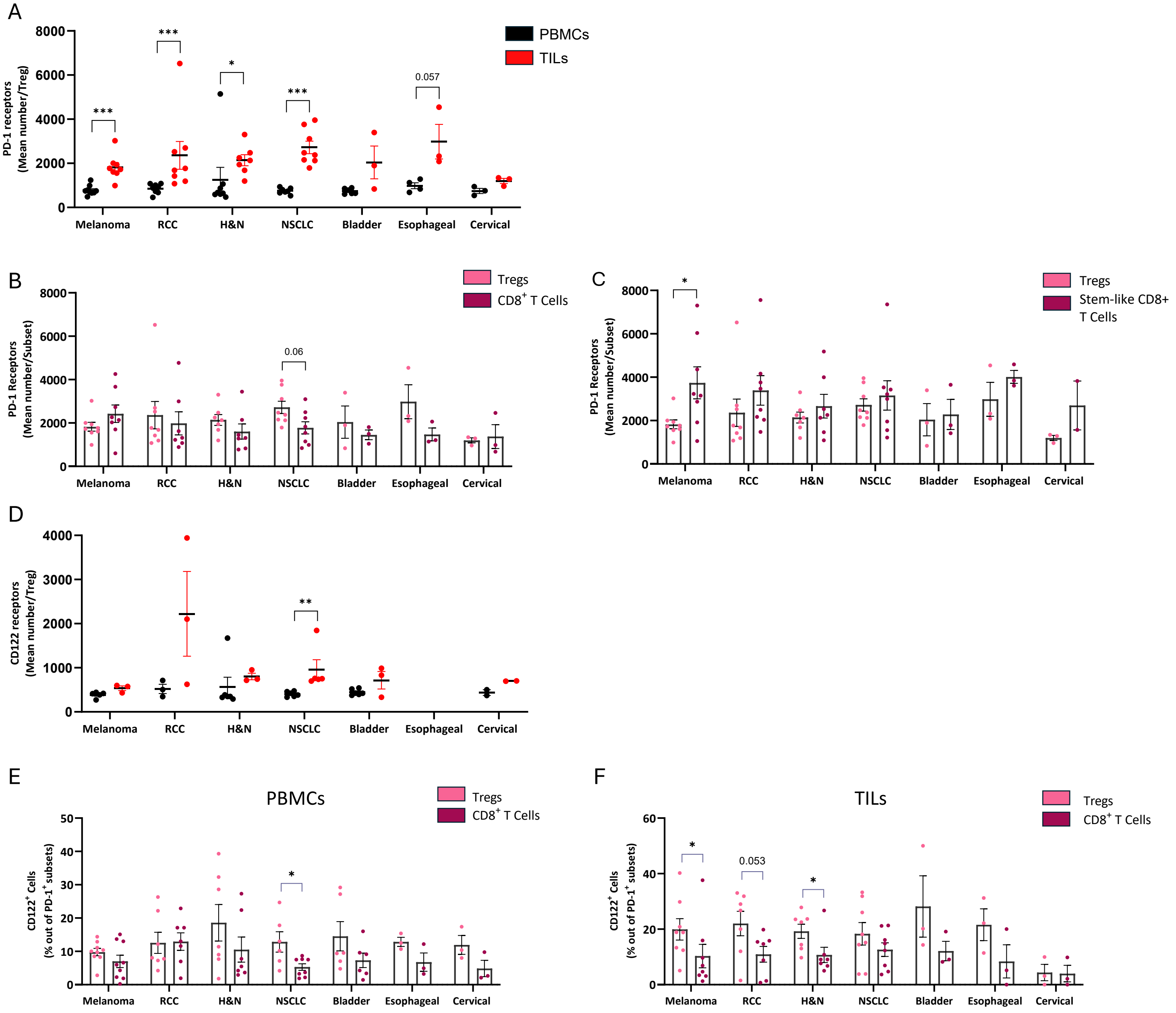

Fig. S2 (A) Absolute quantification of PD-1 receptor number on Tregs. (B) Absolute quantification of PD-1 receptors on Tregs versus total CD8$^+$ T cells in the TIL compartment. (C) Absolute quantification of PD-1 receptors on Tregs versus stem-like CD8$^+$ T cells in the TIL compartment. (D) Absolute quantification of CD122 receptor on Tregs. (E-F) Frequency of CD122$^+$ cells out of PD-1$^+$ Tregs vs PD-1$^+$ CD8$^+$ T cells in (E) PBMCs and (F) TILs. Horizontal lines represent the mean ± SEM; each dot represents an individual patient sample. (A-F). Statistical significance between PBMCs (black) and TILs (red) for each indication as well between Tregs (pink) and total CD8$^+$/Stem-like T cells (purple) within each compartment was determined by nonparametric Mann-Whitney U tests. Horizontal lines represent the mean ± SEM; each dot represents an individual patient sample. Note: Receptor quantification was only performed for subsets containing at least 5 recorded events to ensure signal reliability.

Figure S3

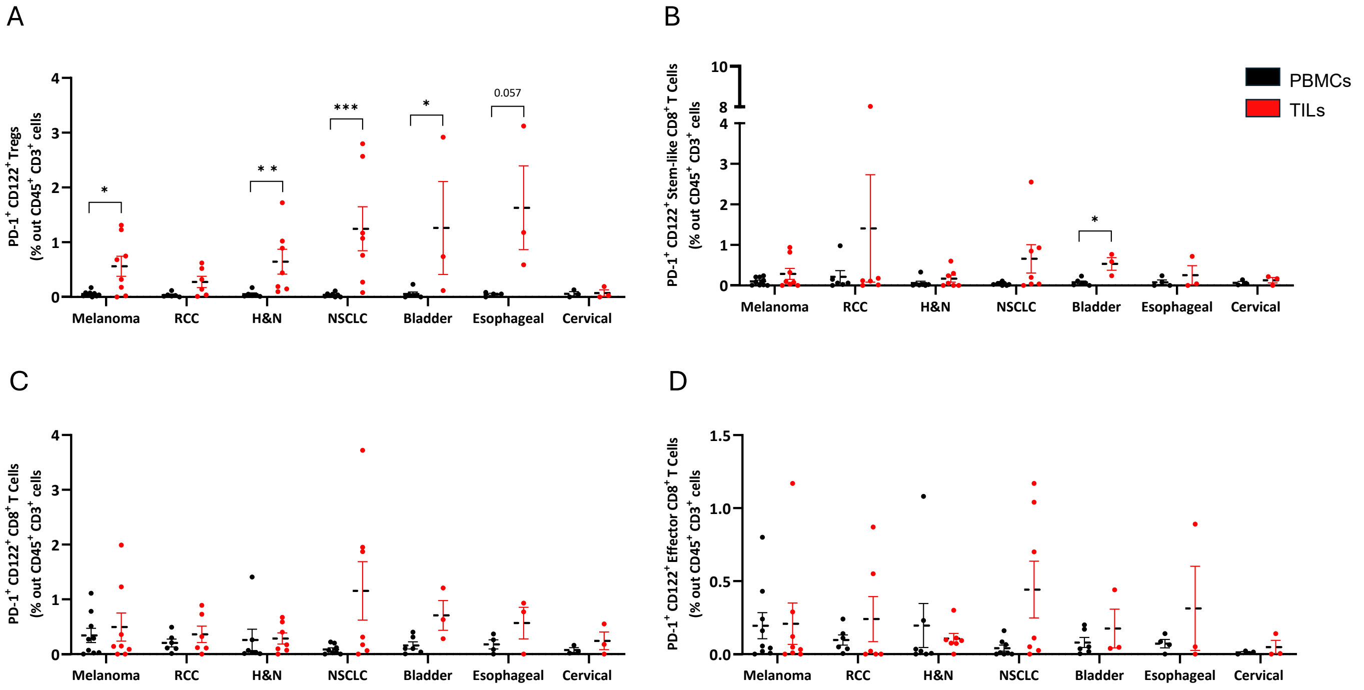

Fig. S3 (A-D) Frequency of PD-1+ CD122+ (A) Tregs, (B) stem-like CD8+ T cells, (C) CD8+ and (D) effector CD8+ T cells out of total CD3+ T cells. PBMCs are depicted in black and TILs in red in all graphs. Statistical significance between PBMCs (black) and TILs (red) for each indication was determined by nonparametric Mann-Whitney U tests. Horizontal lines represent the mean ± SEM; each dot represents an individual patient sample.

Figure S4

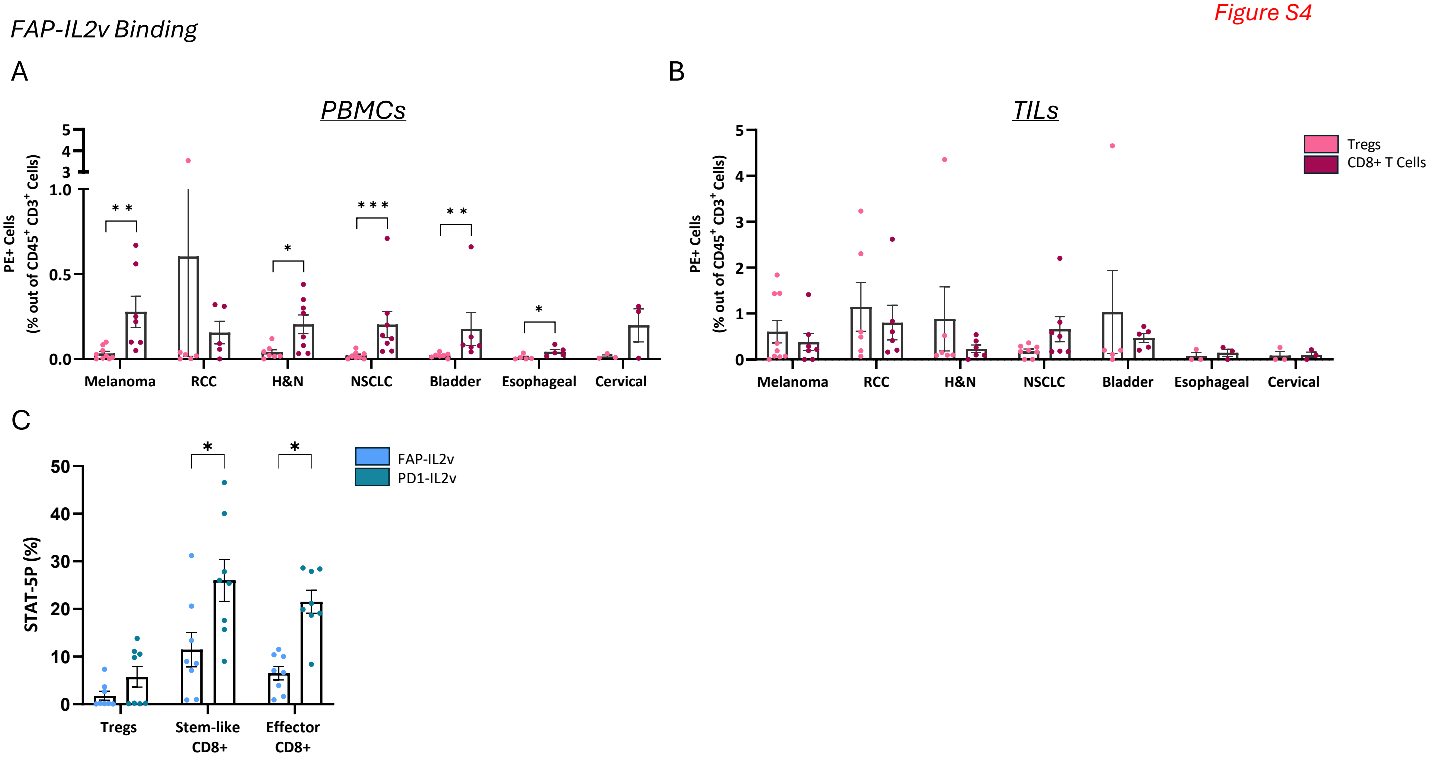

Fig. S4 (A, B) Frequency of FAP-IL2v-bound Tregs (pink) and Total CD8+ T cells (purple) out of total CD3+ T cells in (A) PBMCs and (B) TILs. (C) Frequency of STAT5-P+ cells in Tregs, stem-like and effector CD8+ T cells upon FAP-IL2v (light blue) and PD1-IL2v (turquoise) exposure in HD PBMCs. Statistical significance between Tregs (pink) and total $CD8^+$ T cells (purple) within each compartment as well as FAP-IL2v (light blue) and PD1-IL2v (turquoise)-exposed samples within each subset was determined by nonparametric Mann-Whitney U tests. Horizontal lines represent the mean ± SEM; each dot represents an individual patient sample.
